# Family supportive supervisor behavior and work-family boundary control in teleworkers during a lockdown: Portugal and Pakistan comparison

**DOI:** 10.3389/fpsyg.2022.1008992

**Published:** 2022-09-29

**Authors:** Vânia Sofia Carvalho, Hassan Imam, Maria José Chambel, Mariana Santos

**Affiliations:** ^1^Faculdade de Psicologia, Centro de Investigação em Ciência Psicológica, Universidade de Lisboa, Lisbon, Portugal; ^2^Graduate School of Management, Kyoto University, Kyoto, Japan; ^3^UE Business School, University of Education, Lahore, Pakistan

**Keywords:** family supportive supervisor behavior, telework, lockdown, boundary control, satisfaction with life, COVID-19 pandemic

## Abstract

The imposition of telework due to the COVID-19 pandemic brought with it the need for individuals to readjust their work-non-work boundaries. In this crisis situation, individuals’ needs to manage these boundaries may have been influenced by contextual factors, such as family-supportive supervisor behaviors (FSSB) and macro-structural aspects, such as the country to which the teleworkers belong. This study tests the mediating effect of boundary control on the relationship between FSSB and satisfaction with life and examines the moderating effect of the country (Pakistan vs. Portugal) in the relationship between FSSB and boundary control. With a sample of 108 Portuguese and 118 Pakistani individuals, the results were analyzed using Process tool. FSSB was found to be important for teleworkers to control their boundaries and for their satisfaction with life and this control was also seen to contribute to higher levels of life satisfaction. Differences between the two countries were observed: boundary control mediates the relationship between FSSB and satisfaction with life for Pakistani teleworkers and these workers are more dependent on FSSB to exercise boundary control than Portuguese teleworkers. This study highlights the importance of considering contextual factors when implementing telework. Practical implications are discussed.

## Introduction

Within the context of the COVID-19 pandemic, individuals’ working methods underwent radical changes, namely an abrupt shift to telework (Carnevale and Hatak, 2020). Telework may be defined as a working arrangement away from the conventional workplace, which relies on information and communication technologies for the accomplishment of tasks (Sinclair et al., 2020). In a telework situation, control of the work-family boundary (Clark, 2000) is more easily challenged as workers share the family and workspace, thus making it more difficult to psychologically distance themselves from work and control the boundaries between the two domains (Sinclair et al., 2020). Given that many companies plan to maintain telework in the post-pandemic period, it is important to understand how an organizational context that is conducive to the adoption of this work arrangement may be created.

The boundary theory (Clark, 2000) highlights boundary control, i.e., the ability to decide on how to combine or separate work tasks and family/personal life tasks (Kossek et al., 2012) as one of the important factors for achieving work-family balance. Studies have underlined how this control is essential for employees’ wellbeing since, by allowing individuals to have the power to make decisions on how to balance the performance of their multiple roles, they may feel that they are responding to the most relevant dimensions of their life (Thomas and Ganster, 1995; Thompson and Prottas, 2006).

Despite their importance for effective management of the work-family relationship, few studies have analyzed the factors that can facilitate the control of work-family/personal life boundaries (e.g., Kossek et al., 2012; Capitano et al., 2019). The literature has highlighted family supportive supervisor behavior [family-supportive supervisor behaviors (FSSB), Hammer et al., 2009; Crain and Stevens, 2018] as a key contextual resource for harmonizing this relationship as it enables individuals to manage their family and work responsibilities, preventing stressful situations and ensuring higher levels of wellbeing (Crain and Stevens, 2018). In fact, by manifesting a set of supportive behaviors beyond the work context, such as offering time flexibility to teleworkers, FSSB can be a resource that offers individuals the opportunity to decide on their work and non-work boundaries (i.e., control of their boundaries) (Capitano et al., 2019). Although prior studies (Thomas and Ganster, 1995; Thompson and Prottas, 2006) have already shown the relevance of supervisor support for boundary control, this study has the advantage of analyzing supervisor support for the work-family relationship (FSSB, Hammer et al., 2009), which has an additional effect on the management of these two domains beyond the effect of general supervisor support (Hammer et al., 2009, 2013). Additionally, this study analyzed this effect in the context of telework, i.e., in a context where professional and family roles tend to overlap more and, consequently, are more difficult to manage.

However, although the relationship between FSSB and satisfaction with life or subjective wellbeing is analyzed in a number of studies (e.g., Straub, 2012; Newman et al., 2014; Rathi and Lee, 2017; Yucel and Minnotte, 2017; Shi et al., 2019), there are no studies on the factors that may explain this relationship, especially in a telework context. The teleworking during confinement needs to be framed since for most workers it was an imposition and there was no prior preparation for work in this modality. Thus, teleworkers in this period faced the challenge of dealing with a new way of working and often without specific conditions for this, such as having adequate space. In addition, other household members could be at home at the same time, which can pose added challenges in meeting work and family demands at the same time (Rudolph et al., 2021). Thus, the first objective of this study is to analyze the potential explanatory role that boundary control has in the relationship between FSSB and satisfaction with life in the context of lockdown-induced telework.

Each country is marked by a distinct cultural context (Hofstede, 1980) which has been defined by the literature as a macro-level determining factor in how individuals manage their work and family life (Hammer et al., 2009; Lu et al., 2009; Kossek et al., 2012). Moreover, some studies have pointed to the importance of understanding the specific context of telework in light of cultural patterns (Peters and den Dulk, 2003; Masuda et al., 2012). The analysis of each country, as a consequence of its cultural patterns, has been encompassed in dimensions such as individualism-collectivism, i.e., referring to the extent to which the individual is emphasized over the group in a culture (Hofstede, 1980) and power distance, i.e., the extent to which the less powerful members of institutions and organizations within a country expect and accept power to be distributed unequally (Hofstede, 1980). In general, it is argued that telework will be more easily implemented in countries with individualistic cultural contexts and with more power decentralization since independence and autonomy are regarded as core values (Peters and den Dulk, 2003; Masuda et al., 2012). The Global Leadership and Organizational Effectiveness (GLOBE) project by House and colleagues (House et al., 2004) validates Hofstede’s (1980) typology and identifies these and other dimensions to distinguish specific aspects of different countries’ cultures. This study collected data in two countries with distinct cultural patterns: Portugal and Pakistan. According to GLOBE, Portugal is a more individualist country with more decentralized power while Pakistan is a more collectivist country with more centralized power. The study by Lu et al. (2009) shows how supervisors’ emotional support in employees’ family life may be more important for countries with a collectivist culture when compared to an individualist culture. Thus, in a context of imposed telework due to lockdown, where supervisors needed to redefine their support and employees their work-family boundaries, different reactions would be expected according to the country in question. Thus, the second objective of this study is to analyze the extent to which belonging to two countries (Portugal vs. Pakistan) with different cultures may condition the relationship between FSSB and the perception of boundary control.

This study offers several contributions to the theory and organizational management of the work-family interface. Firstly, it should be noted that the data collected for this study, namely through workers in lockdown and, consequently, in telework, may provide important knowledge for action in crisis contexts. More specifically, it may contribute to an understanding of the role of the supervisor and boundary control as important resources in this context of telework and subsequently lead to the establishment of practical action strategies. Secondly, from a theoretical point of view, this model emphasizes the potential effect that a contextual variable, namely FSSB, may have on an individual variable, which has not been studied extensively in the literature on the work-family relationship, i.e., boundary control (Clark, 2000). Furthermore, the comparison between Portugal and Pakistan will allow for a better understanding of the realities of these two countries with regard to telework, FSSB and boundary control, thus contributing to the design of more tailored intervention strategies. Overall, cross-cultural studies help enhance international understanding, encourage collaboration, and improve communication (Nadeem and de Luque, 2018), which is also the aim of this study.

## Theoretical framework

### The mediation role of boundary control

Telework has long been termed a family-friendly practice, therefore associated with benefits such as enhanced work-family balance afforded by the flexibility to balance the two domains and more autonomy in the management of work tasks (Sardeshmukh et al., 2012). However, prior to the pandemic, many companies had not yet implemented this practice and it thus emerged as an imposition for which employers and workers were not prepared (Desilver, 2020; Sinclair et al., 2020). In fact, the abrupt shift to telework during lockdown had distinct contours. Firstly, telework is a measure regarded as voluntary, however, during lockdown it became mandatory (Sinclair et al., 2020). In addition, some factors may have hindered telework during lockdown, such as couples’ dual employment where they were both teleworking and their children were also at home in a situation of distance learning due to the closure of schools. Thus, many were forced to respond to the demands of work and support their children simultaneously (Rudolph et al., 2021). Moreover, the differentiation of workspace and time has traditionally served to configure the different roles played by individuals (Clark, 2000). A typical example is that of a worker performing work tasks in the workspace for a specific period of hours (e.g., Monday–Friday, 9–5) who is physically absent from the workplace when involved in non-work tasks such as during the evenings and weekends (Allen et al., 2014). Therefore, it may be said that during the lockdown period telework caused not only the absence of physical boundaries but also temporal boundaries which, in turn, leads teleworkers to be constantly thinking about work or performing professional tasks beyond the actual work schedule (Grant et al., 2013).

The telework phenomenon during lockdown may be understood by considering how the boundaries between the work and family (personal life) domains are managed by individuals in order to achieve balance (Clark, 2000). Boundaries may be physical, temporal or psychological and are influenced by flexibility, i.e., the extent to which spatial and temporal boundaries are pliable, and permeability, i.e., the extent to which a person physically located in one domain may be psychologically or behaviorally engaged in another domain. According to Clark (2000) and Kossek et al. (2012), the effective management of these boundaries depends on the extent to which individuals feel able to control them. For example, for individuals to prevent work from invading their family life, it is fundamental that they feel in control of their leisure time and can turn off their professional mobile phone to avoid being contacted (e.g., by supervisors, colleagues or clients). Likewise, to prevent family from invading their professional life, it is equally crucial that individuals feel they can control their thoughts and worries when they are working, to concentrate solely on performing their professional tasks. In fact, it is this control that enables individuals to behave according to their preferences and the demands of their roles in these two domains: high levels of control translate into congruence between their behaviors, preferences and/or role demands, while the opposite occurs when control is low (Capitano et al., 2019).

For the above reasons, during lockdown boundary control naturally took on particular relevance for workers’ wellbeing. In fact, control over the time, frequency and direction of boundary transitions between the work and family spheres is an important resource for individuals that will help them to effectively manage the various roles and, consequently, develop feelings of self-efficacy (Kossek and Lautsch, 2012). Moreover, it is when individuals feel they have control over the work-family boundaries that they perceive an alignment with their identity and values (Kossek, 2016) and obtain satisfaction from the performance of their life roles (Capitano et al., 2019), thus achieving high levels of wellbeing.

Satisfaction with life represents one of the indicators of subjective wellbeing and may be defined as a cognitive process characterized by individual judgment on quality of life in terms of self-imposed criteria (Diener et al., 1985; Pavot and Diener, 1993). People report high satisfaction with life when their life circumstances are in line with these criteria (Pavot and Diener, 1993). Thus, boundary control is expected to be a relevant variable to explain the extent to which teleworkers are satisfied with their life.

On the other hand, the boundary theory (Clark, 2000) highlights that individuals’ management of the work-family boundaries is dependent on situational factors, namely *border-keepers*, among whom direct supervisors are particularly relevant (Park et al., 2011). In fact, supervisors may display varying degrees of flexibility whether by adapting professional conditions to each individual’s family situation or encouraging/discouraging them to use family support policies and practices.

FSSB may be observed through the family support behaviors adopted by the supervisor in order to help employees balance their work and family lives (Hammer et al., 2009). These behaviors, divided into emotional, instrumental support behaviors, role modeling and creative management of the work-family relationship, may be important for individuals to feel that they control the boundaries between the domains. For example, emotional support is when individuals feel their needs are being taken into consideration and that they can communicate with the source of support whenever necessary (Hammer et al., 2009), which may generate the feeling of support from their supervisor to adapt/modify their work schedule. In turn, role-modeling behaviors are related to how supervisors provide examples of strategies and behaviors that foster the effective integration of work and family responsibilities (Hammer et al., 2009). Therefore, if supervisors display flexible boundary-adjusting behaviors, their employees will also be more encouraged to do so. Instrumental support refers to how the supervisor responds to employees’ specific needs regarding the work-family relationship by providing services or resources so that they can effectively manage their responsibilities in these two domains (Hammer et al., 2009). More specifically, if an individual needs to deal with a family demand during working hours, the supervisor can work with the team to readjust the worker’s schedule to meet that need. Finally, creative management of the work-family relationship, which is more proactive and strategic in nature, involves restructuring work to facilitate workers’ effectiveness (Hammer et al., 2009). This creative management may involve, for example, the use of a collaborative platform to facilitate communication among team members in a telework arrangement, facilitating not only the performance of the professional activity, but also the adjustment to each worker’s family/personal life by avoiding excessive meetings.

Several studies have corroborated the beneficial effect of supervisor support for both work-family boundary management and workers’ wellbeing (Crain and Stevens, 2018). For example, the study by Thomas and Ganster (1995) showed that supportive practices, including supervisor support, increased the perception of control over work and family matters and that this perception of control translated into lower levels of work-family conflict, job dissatisfaction, depression, somatic complaints, and blood cholesterol. Thompson and Prottas (2005) also showed that supervisor support was beneficial for individuals to increase their perception of control over the work-family boundaries and that this perception was fundamental for satisfaction with life. Although supervisor support was not geared specifically toward the work-family relationship in these studies, and they were not conducted in a context of telework, they still offer consistency to the following hypothesis:

H1: The relationship between FSSB and satisfaction with life in lockdown-induced telework is mediated by perceived control of the work-family boundaries.

### The moderating role of country (Portugal and Pakistan) in the family-supportive supervisor behaviors and boundary control relationship

Several authors have highlighted the importance of each country’s culture, not only in relation to how people balance their work and family (Powell et al., 2009; Allen et al., 2015) but also in the adoption of organizational practices that allow workers to establish a balance between their work and family life, namely telework (Peters and den Dulk, 2003; Masuda et al., 2012).

The culture of each country is characterized as a set of beliefs, values and norms shared by individuals with a common historical experience, and which influence their behavior (Hofstede, 2005). Two dimensions of this culture have been highlighted as influencing the adoption of telework: collectivism/individualism and power distance. In an individualist culture, behaviors and beliefs are mostly determined by the person, whereas in collectivist cultures loyalty to the group has the strongest influence on individuals’ behaviors (Hofstede, 1980). Thus, as telework restricts the daily and direct contact between worker-supervisor and worker-co-workers, this work arrangement is less likely to be adopted in collectivist cultures (Gajendran and Harrison, 2007). Moreover, in countries with collectivist cultures, workers tend to place more value on the roles played within the family context and feel they should spend more time in the family setting (Aryee et al., 1999; Masuda et al., 2012). Thus, the imposition of telework in countries with a collectivist culture can create a paradoxical situation, as workers are at home in the space usually dedicated to the family domain which they value most, but with the obligation of performing their professional role. Conversely, telework is likely to be more frequently adopted by organizations in countries with an individualist culture (Masuda et al., 2012). Since this work arrangement is associated with greater employees’ autonomy (i.e., control over when and how to perform work tasks and work-family balance choices), it is more accepted in an individualist culture (Peters and den Dulk, 2003; Masuda et al., 2012). Gajendran and Harrison (2007) also argue that the adoption of telework implies workers having suitable conditions, such as technological support and physical space (e.g., office) to work in their homes. In more collectivist countries, homes tend to be shared by more family members and there is a greater likelihood of boundary blurring, which makes it more difficult to manage the work-family boundaries (Masuda et al., 2012).

As far as power distance is concerned (Hofstede, 1980), its presence implies high power centralization among few people and many layers of supervision in vertical hierarchies, hindering the adoption of telework (Peters and den Dulk, 2003) since as already mentioned, this work arrangement fosters workers’ autonomy, allowing them to make decisions.

In light of the abrupt shift to telework in the context of the pandemic and considering the aforementioned factors, it may be inferred that for Portugal (more individualistic and less distant from power) compared to Pakistan (more collectivistic and more distant from power) (Nadeem and de Luque, 2018; GLOBE, 2020) this change was more easily adopted by workers. Thus, in comparison with Pakistan, so much dependence on supervisor support for the work-family relationship so that workers can adjust the time, frequency and direction of their transitions (i.e., boundary control) between the two domains is less likely in Portugal. On the other hand, in Pakistan, as the culture places greater importance on group dependency and power is more centralized, the implementation of telework is likely to be more difficult and consequently there will be greater dependence on the support of the supervisor for workers to be able to control their establishment of work-family boundaries. Therefore, as an illustrative example, in the situation of a sudden shift to telework due to COVID-19, the Portuguese teleworker may have taken the freedom to choose work/non-work time boundaries more autonomously while the Pakistani worker may have needed prior approval from his or her supervisor to do so. The study by Lu et al. (2009) found that FSSB was more important in helping workers to balance their work and family life in collectivist cultures. More specifically, it was found to have a more mitigating effect on work-family conflict in Taiwan (collectivist) than in the United Kingdom (individualist).

In view of the above, it was established that:

H2: The relationship between FSSB and boundary control is moderated by the country, to the extent that this relationship is significantly stronger for Pakistani teleworkers compared to Portuguese teleworkers.

## Materials and methods

### Procedure

This study was disseminated by the Human Resources department of several companies in the service sector, both in Portugal and Pakistan, which shifted to full-time telework during the first COVID-19 lockdown. The snowball method was also used to obtain participants for both samples. The questionnaire was approved by Ethics Committee of Faculty of Psychology, University of Lisbon. Participation in the study was voluntary and participants were guaranteed anonymity. In both countries data were collected between 15 March and 15 April, 2020, through participants’ responses to a questionnaire composed by 20 questions in total and with two sections—a first section with demographic questions and a second section of questions structured with scales described below. The questionnaire was available on the Survey Monkey platform.

### Sample

The sample consisted of 226 workers from various areas who were teleworking due to lockdown. Of these workers 108 (47.8%) were Portuguese and 118 (52.2%) Pakistani. The sample was non-probability and was composed of 55.3% female workers (Portugal: 63%; Pakistan: 48.3%).

### Measures

#### Family supportive supervisor behaviors

This variable was measured through 8 items (FSSB; Hammer et al., 2009) scale (e.g., My coordinator/direct supervisor has been concerned about my wellbeing and I have been able to rely on my coordinator/direct supervisor to help me solve conflicts between my professional and personal/family tasks). The participants were asked to rate each item on a Likert scale ranging from 1 (Strongly Disagree) to 5 (Strongly Agree), with high scores on these scales indicating high levels of supervisor support. This variable revealed good internal consistency, both for the Portuguese and Pakistani samples (α = 0.94 and 0.91, respectively).

#### Boundary control

Three items from the Boundary Control scale (Kossek et al., 2012) were used (e.g., I have controlled whether I am able to keep my work and personal/family life separate and I have controlled how I combine my work and personal/family life activities throughout the day). Participants were asked to rate each item on a Likert scale ranging from 1 (Strongly Disagree) to 5 (Strongly Agree). Thus, high scores on these scales indicated high levels of individuals’ perception of boundary control. This scale revealed an internal consistency of 0.81 for the Portuguese sample and 0.74 for the Pakistani sample.

#### Satisfaction with life

The Satisfaction with Life Scale (SWLS; Diener et al., 1985) was used to measure this variable. This scale had previously been adapted and validated for the Portuguese population (Neto et al., 1990; Simões, 1992) and had also been used in Pakistan (Naseem, 2018). This 5-item scale (e.g., In many ways (my life) is close to my ideal and If I could live my life again, I would barely change anything), revealed an internal consistency of 0.79 for the Portuguese sample, and 0.80 for the Pakistani sample. The participants responded using a 7-point Likert scale (1 = Strongly disagree to 7 = Strongly agree).

#### Control variables

Previous studies suggest that there may be differences in results depending on the gender of participants as far as FSSB (Huffman and Olson, 2017) and boundary control (Straub et al., 2019) are concerned. Thus, to avoid alternative explanations for the results, the gender of the participants was controlled, coded into a categorical variable for statistical purposes, where 0 = Female; 1 = Male. Furthermore, the results may also be affected by workers having children or not, both at the level of FSSB (Hammer et al., 2009) and at the level of boundary control (Mellner et al., 2014).

In Pakistan, the scales in the original English version were used while in Portugal the Portuguese version was used, and the Brislin method (1980) was used in the translation of those with no previous version.

## Data analysis

First, due to the fact that all the measures were assessed as self-reports, a confirmatory factor analysis was conducted to examine whether the measures indeed represented different constructs. Confirmatory factor analyses (CFA) (Brown, 2015), with structural equation modeling were implemented with Mplus 7.2 (Múthen and Múthen, 1998–2015). The maximum likelihood estimation provides the well-known global fit statistics for structural equation modeling methods: comparative fit index (CFI; satisfactory values of 0.90 and above), Tucker–Lewis index (TLI; satisfactory values of 0.90 and above) and root mean squared error of approximation (RMSEA; satisfactory values below 0.08) (van de Schoot et al., 2012).

The measures of central tendency and dispersion and the internal consistency indices (i.e., Cronbach’s alpha) were then calculated for the variables under study, as well as Pearson’s correlations between all the variables (Table 1). Finally, hypothesis testing was conducted, using the SPSS Process tool, where the proposed mediation and moderation were analyzed. More specifically, Process model 7 was used (Hayes, 2012), which tests a mediated moderation model. The bootstrapping method (5,000) was also used, a non-parametric method based on resampling, which is repeated multiple times, and makes it possible to estimate the distribution of the sample in terms of direct and indirect effects (Bollen and Stine, 1990).

**TABLE 1:** Mean, standard deviation (SD) and correlations (*N* = 226).

	Portugal						Pakistan					
		
	Média	DP	*R*				Média	DP	*r*			
						
			1.	2.	3.	4.			1.	2.	3.	4.
1. Sex^a^	–	–					–	–				
2. Children	–	–	–0.29**				–	–	0.20*			
3. FSSB	3.36	0.82	0.08	0.09			3.08	0.83	–0.11	0.05		
4. Boundary control	3.47	0.82	0.11	0.08	0.08		3.11	0.91	0.03	0.01	0.33**	
5. SWL	3.49	0.66	0.11	–0.18	0.10	0.19*	3.38	0.73	–0.14	–0.17	0.33**	0.17

^a^Dummy variable (0 = women and 1 = men). *ρ < 0.05; **ρ < 0.001. FSSB, Family Supportive Supervisor Behavior; SWL, Satisfaction with Life.

## Results

### Sample description

As aforementioned, the sample of this study was non-probability and was composed of 55.3% female workers (Portugal: 63%; Pakistan: 48.3%). Regarding marital status, most of the workers were married or in a stable union (55.3%—Portugal: 49.1%; Pakistan: 61%) and 50% (Portugal: 41.7%; Pakistan: 57.6%) of the respondents had children. Finally, prior to lockdown, most of the workers (64.6%—Portugal: 53.7%; Pakistan: 78%) had never experienced a telework situation, 18.6% (Portugal: 27.8%; Pakistan: 10.2%) worked 1 day a week from home, 8% (Portugal: 10.2%; Pakistan: 5.9%) worked 1 or 2 days a week from home and 4.9% (Portugal: 4.6%; Pakistan: 5.1%) were teleworking all week.

### Confirmatory factor analysis

The theoretical model comprising the FSSB, boundary control and satisfaction with life latent variables proved to be adequate [χ^2^(102) = 218.19, *p* < 0.001; TLI = 0.60; CFI = 0.65; RMSEA = 0.07; SRMR = 0.07]. When comparing the theoretical model with the one-factor model, the fit indices were found to be lower and below the threshold in the one-factor measurement model (IFI = 0.67; TLI = 0.61; CFI = 0.66; RMSEA = 0.16; SRMR = 0.16), compared to the theoretical measurement model. Furthermore, the chi-square of the one-factor model proved to be significantly higher [χ^2^(104) = 701.75, *p* < 0.001], and the difference between the two models was significant [Δχ^2^(2) = 483.56, *p* < 0.001]. Taking this into account, it may be assumed that the theoretical measurement model is more suitable for the analysis of the data in the two samples.

### Hypothesis testing

The first hypothesis of this study proposed a mediating effect of boundary control on the relationship between FSSB and satisfaction with life. As may be seen in Tables 2, 3, the relationship between FSSB and boundary control is positive and significant (*B* = 0.37, *p* < 0.001) and the relationship between boundary control and satisfaction with life is also positive and significant (*B* = 0.12, *p* < 0.001), and there is also a positive and significant direct relationship between FSSB and satisfaction with life (*B* = 0.17, *p* < 0.001). When analyzing the indirect effects values, it was found to be 0.05 for Pakistan, which is significant (CI = [0.00, 0.10]), and 0.01 for Portugal, which is non-significant (CI = [–0.03, 0.04]). Thus, Hypothesis 1 was partially supported.

**TABLE 2:** Mediation and moderation analysis of studied variables (*N* = 226).

	Boundary control (*R*^2^ = 0.34; *p* < 0.001)				Satisfaction with life (*R*^2^ = 0.34; *p* < 0.001)			
		
	*B*	SE	*T*	*p*	*B*	SE	*t*	*p*
FSSB	0.37	0.09	3.95	0.0001	0.17	0.05	3.19	0.0016
Boundary control	–	–	–	–	0.12	0.05	2.40	0.0170
Country	1.32	0.45	2.90	0.0040	–	–	–	–
FSSB*Pakistan	0.37	0.09	3.95	0.0001	–	–	–	–
FSSB*Portugal	0.06	0.10	0.56	0.58	–	–	–	–

**TABLE 3:** Analysis of conditional indirect effects of FSSB on satisfaction with life.

Mediator (boundary control)	Satisfaction with life		
	
	*B*	Boot SE	IC (95%, bias-corrected bootstrap)
Pakistan	0.05	0.02	[0.00, 0.10]
Portugal	0.01	0.02	[–0.03, 0.04]

As regards the moderating effect of country on the relationship between FSSB and boundary control, the interaction was found to be significant for the Pakistani sample (*B* = 0.37, CI = [0.19, 0.56]), but not for the Portuguese sample (*B* = 0.06, CI = [–0.13, 0.25]). The indirect effects are significant in the case of Pakistan (*B* = 0.05, CI = [0.00, 0.09]) but not for the Portuguese sample (*B* = 0.01, CI = [–0.02, 0.00]). The moderate mediation index was not found to be statistically significant (Index = 0.04, CI = [–0.10, 0.00]). Thus, although moderate mediation was not found for the two countries, moderation was observed in the case of the Pakistani sample (as was the mediation).

**FIGURE 1:**
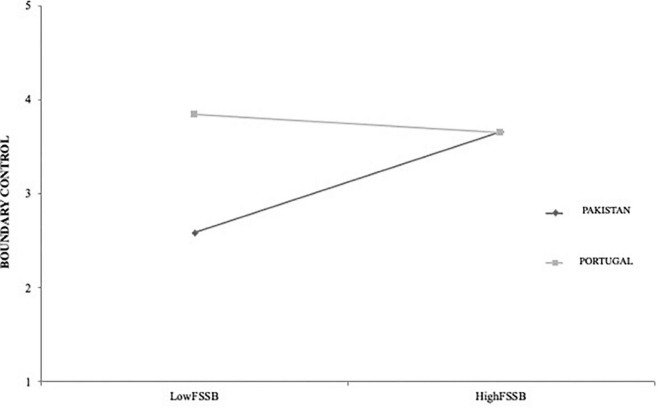
The moderated role of country in the relationship between FSSB and Boundary Control.

Figure 1 shows that as far as the Pakistani is concerned, boundary control is higher when there is a higher level of support from the FSSB, However, the same is not observed for the Portuguese culture, where boundary control remains practically the same, regardless of the level of FSSB. Thus, the Pakistani culture appears to increase the impact of supervisor support on the work-family relationship, since when this support is high, the boundary control is significantly higher. Therefore, through this positive effect, it may be inferred that the Pakistani culture strengthens the relationship between supervisor support for the work-family relationship and boundary control, and this relationship is not so culture-dependent for Portuguese workers. Thus, Hypothesis 2 is supported.

## Discussion

This study examined the mediating role of boundary control in the relationship between family supportive supervisor behavior (FSSB) and satisfaction with life among teleworkers during lockdown. As expected, the results suggest that FSSB is important for teleworkers to control their boundaries and, in turn, this control is important for teleworkers to assess their lives positively. Moreover, in a direct manner, FSSB also contributes to this positive evaluation of the life of teleworkers. When analyzing the indirect effect, it was found to be significant only for Pakistan, i.e., mediation only occurred in this country. Furthermore, the moderating role of the country in the relationship between FSSB for the work-family relationship and boundary control was analyzed and showed that, as expected, FSSB had a more prominent role in the boundary control of the Pakistani teleworkers with a more collectivist culture and a greater distance to power.

This result emphasizes the importance of boundary control for teleworkers, since it grants them the freedom to harmonize their behaviors and/or role preferences/requirements (Clark, 2000; Kossek et al., 2012; Capitano et al., 2019). This boundary control proved to be an important factor for individuals to positively evaluate their lives, as shown in other studies, albeit not focused on teleworkers (Piszczek, 2017; Straub et al., 2019). Hence, especially in the context of lockdown due to COVID-19, when telework was imposed without previous preparation for many employees and in a situation of lack of work conditions to teleworkers (e.g., children at home, lack of space) (Sinclair et al., 2020; Rudolph et al., 2021), the boundary control was crucial to maintain healthy workers. On the other hand, the results of this study point to FSSB as a relevant contextual variable for the achievement of this boundary control, which is in line with the Boundary Theory (Clark, 2000), more specifically due to the importance this theory attributes to border keepers in boundary management. Moreover, beyond its direct weight in boundary management, FSSB has a positive effect on teleworkers’ satisfaction with life. Although not focusing on teleworkers, prior studies have also shown the direct relationship between FSSB and satisfaction with life (e.g., Straub, 2012; Shi et al., 2019).

Despite the afore-mentioned relationships being significant, it should be noted that the mediation effect was not observed for the Portuguese workers, which appears to suggest that boundary control is not so important to explain the impact of supervisor support on satisfaction with life among these workers. A possible explanation may be that individuals perceive the organization where they work as having a “family-friendly” culture and therefore satisfaction with life is only dependent on FSSB and not so much on how individuals control their work-family boundaries. Supervisor support is therefore a more salient feature. This may occur due to the fact that the perception of family support on the part of the organizations and FSSB are related and have been highlighted as key antecedents to work-family balance (Mills et al., 2014).

When analyzing the moderating effect of the country (Portugal vs. Pakistan) on the relationship between FSSB and boundary control, FSSB was found to be essential for the Pakistani teleworkers, which was not the case for the sample of Portuguese workers. This result is in line with the idea that for countries with more collectivist cultures, adaptation to telework is more complex and more role blurring may be created (Masuda et al., 2012). Pakistani teleworkers with collectivist values may be more dependent on their work group (Hofstede, 1980) and the sudden shift to telework may have caused greater disruption since this work arrangement implies working alone. Moreover, for Pakistani teleworkers, supervisor support proved to be crucial, which is in keeping with the idea that these workers are less autonomous in decision making and need more supervisor support to define when, where and how they can transition across boundaries. At the same time, this study shows how the need for supervisor support/approval in countries with cultures with a greater power distance (Hofstede, 1980) may be more accentuated. In contrast, Portuguese workers, belonging to a more individualist culture, assert their greater boundary control autonomy and are thus not so dependent on supervisor support. Although the study by Lu et al. (2009) did not focus on teleworkers or boundary control, it also showed how the supervisor’s emotional support for the work-family relationship was a variable to which a collectivist country (Taiwan) attached more importance in order to reduce work-family conflict, when compared to a more individualist country (i.e., United Kingdom).

## Limitations and future studies

This study has some limitations. Firstly, the fact that the study is cross-sectional only provides information on the positive or negative nature between the variables and their statistical significance and not necessarily the existence of a causal relationship between them. In order to analyze the latter, a longitudinal study would need to be conducted. Furthermore, it might have been interesting to have collected data at the beginning and end of the lockdown period to ascertain whether there were any changes in the two countries, namely in relation to supervisor support and boundary control. Another limitation is related to the fact that the data were collected by means of a questionnaire that assessed the individuals’ perceptions. Thus, the data obtained are subjective and may be subject to bias and social desirability, despite the fact that anonymity was guaranteed, and therefore may not correspond to reality. In order to overcome this limitation, several sources could be used for comparison and for a better understanding of the reality under study. Additionally, given the small sample size (*N* = 226), it is not possible to generalize the results. Furthermore, this sample is composed exclusively of individuals in a telework situation due to lockdown and it would therefore be interesting in future studies to conduct research involving other conditions (for example, individuals who work in a face-to-face regime, or teleworking under normal conditions), in order to compare the results.

Furthermore, although a moderating effect of the country was found, another limitation of this study is the fact that the cultural differences between Portugal and Pakistan in terms of collectivism/individualism and power distance are not very marked. However, the values for the dimensions used to justify the cultural differences between Portugal and Pakistan, i.e., collectivism/individualism and power-centeredness, may not correspond to the specific reality of the individuals who participated in the study. Thus, questions related to these dimensions could be included in the questionnaires to obtain more reliable data, and a highly diversified sample would be required. It might also be interesting to conduct a study with other countries with more contrasting cultural values. Another limitation of this study is the fact that no distinction was made between the individuals who had previous telework experience and those who only adopted this work arrangement as a result of pandemic-induced telework. Therefore, it would be interesting in future studies to ascertain the impact this factor may have had on supervisor support for the work-family relationship and on boundary control.

## Practical implications

Despite the above-mentioned limitations, some of the findings’ practical implications for the organizational context may be highlighted. This study confirms the important role of supervisor support for the work-family relationship and of boundary control for teleworkers’ wellbeing, i.e., satisfaction with life. Firstly, the direct effect of supervisor support for the work-family relationship on boundary control highlights the importance of supervisors considering the needs and demands of employees outside the workplace, especially in telework, where they can be more difficult to identify if there is no effective communication. To this end, as suggested by Perrigino and Raveendhran (2020), supervisors should identify the needs and preferences of their employees in order to work with them to adjust the temporal and psychological boundaries between work and personal life in light of their differences.

On the other hand, the mediating role of boundary control highlights the importance of implementing practices that ensure greater boundary control for all employees, not forcing a specific boundary management strategy, as employees will experience greater wellbeing if they are free to control their own boundaries between work and family, as opposed to responding to supervisor pressure (Piszczek, 2017). Furthermore, this research highlights the importance of training supervisors in the use of supportive work-family relationship behaviors, as they can be essential for employees to control boundaries. In this regard, Mills et al. (2014) state that the mere existence of training is able to promote a positive work-family climate, even before the learned techniques are practically transferred to the work context.

Additionally, this study also shows that macro contextual factors need to be considered when seeking to design better solutions for teleworkers, namely the culture of each country. More specifically, organizations’ design of family support mechanisms should reflect the cultural values of the country in question (Peters and den Dulk, 2003; Masuda et al., 2012).

Due to the increased prevalence of telework triggered by the pandemic, which implies a greater distance between employees and supervisors, organizations should invest in promoting the wellbeing and satisfaction of their employees, using the practices suggested in this study.

## Data availability statement

The raw data supporting the conclusions of this article will be made available by the authors, without undue reservation.

## Ethics statement

The studies involving human participants were reviewed and approved by Comissão de Deontologia da Faculdade de Psicologia da Universidade de Lisboa. Written informed consent for participation was not required for this study in accordance with the national legislation and the institutional requirements.

## Author contributions

VC involved in writing, analysis, and editing process. HI involved in data collection and revision process. MC involved in the conceptualization, data collection, and original draft preparation. MS involved in the first draft of the article. All authors contributed to the article and approved the submitted version.
